# Health Inequality among Fishery Workers during Climate Change: A National Population-Based and Retrospective Longitudinal Cohort Study

**DOI:** 10.3390/ijerph191610281

**Published:** 2022-08-18

**Authors:** Ming-Shyan Lin, Yu-Chih Lin, Tung-Jung Huang, Mei-Yen Chen

**Affiliations:** 1 Division of Internal Medicine, Department of Cardiology, Chang Gung Memorial Hospital, Chiayi 613, Taiwan; 2 Department of Nursing, Chang Gung University of Science and Technology, Chiayi 613, Taiwan; 3 Department of Family Medicine, Chang Gung Memorial Hospital, Chiayi 613, Taiwan; 4 Department of Pulmonary Disease and Critical Care, Chang Gung Memorial Hospital, Yunlin 638, Taiwan; 5 School of Nursing, Chang Gung University, Taoyuan 333, Taiwan

**Keywords:** fishery workers, propensity score-matched, Cox proportional hazard model, cardiometabolic diseases, chronic viral hepatitis

## Abstract

Background: Owing to specific working environments, it is important to attain sustainable development goals for the health of fishery workers during climate change. Fishery workers have a hazardous working environment, leading to specific injuries and fatal events. However, limited studies have investigated the health status of fishery workers through long-term longitudinal follow-up and compared it with that of farmers and employed workers with similar socioeconomic status. Methods: The Longitudinal Health Insurance Database 2000, a subset of the Taiwan National Health Insurance Research Database was used for this retrospective cohort study. Only fishery workers, farmers, and employed workers were included. Based on the majority of causes of death and related diseases, participants newly diagnosed with 18 diseases, classified into cardiometabolic diseases, mental illness, chronic kidney disease, infection, and malignancy, were included. Participants with an old diagnosis of these diseases were excluded. All included participants were followed up from 1 July 2000 to the diagnosis and withdrawal date, or 31 December 2012, whichever occurred first. Due to the substantial difference in the baseline demographics, we executed a cohort study with propensity score-matched and applied the Cox model to explore the participants’ health status. Results: After matching, there were negligible differences in the baseline demographics of fishery workers, farmers, and employed workers. Compared to farmers and employed workers, fishery workers were more frequently diagnosed with 11 and 14 diseases, respectively, such as hypertension (hazard ratio [HR]: 1.11, *p* < 0.01), diabetes (HR: 1.21, *p* < 0.001), dyslipidemia (HR: 1.18, *p* < 0.001), depression (HR: 1.38, *p* < 0.001), peptic ulcer (HR: 1.17, *p* < 0.001), chronic viral hepatitis (HR: 2.06, *p* < 0.001), hepatocellular carcinoma (HR: 1.67, *p* < 0.001), and total malignancy (HR: 1.26, *p* < 0.001). Conclusions: Compared to farmers and employed workers, fishery workers were more impacted by cardiometabolic diseases, mental illness, infection, and malignancy. Therefore, it is imperative to specifically focus on health policies for fishery workers, such as providing curable antiviral treatment and initiating culture-tailored health promotion programs, to mitigate health inequality.

## 1. Introduction

In Taiwan, 130,000 households, with approximately 340,000 people, are engaged in the fishery industry (also known as aquaculture farming), including inland and marine farming. According to an official report [1], the average annual fisheries production is >1.27 million tons, with a value of approximately 32 million USD. Since Taiwan is situated in a subtropical region, its marine fisheries are well developed. Due to pioneering aquaculture techniques, >100 aquatic species are farmed; therefore, Taiwan has won the reputation of an “aquaculture kingdom” [1]. However, an increased number of fishery workers have been leaving fish farming due to personal health issues, aging, and reducing fish production due to climate change [2,3]. Based on the sustainable development goals set by the United Nations [4], it is crucially important to continuously ensure sufficient food and safety and promote the health of fishery workers.

According to previous cross-sectional studies from Asia, Australia, Brazil, Europe, and the United States, fishery workers tend to have physical and mental illnesses, including cardiometabolic diseases, musculoskeletal injuries, heat stress, respiratory symptoms, infection, and depression owing to extreme temperature events and performance of hazardous tasks [5,6,7,8,9]. Most previous studies on the health of fishery workers have used short-term and cross-sectional study designs; however, limited studies have used long-term and longitudinal cohort study designs. Furthermore, the health and well-being of millions of fishery workers have been neglected in many countries [10].

Malignancy and stroke, hypertension, coronary heart disease, and diabetes (also called cardiometabolic diseases) have been the major causes of death for many years in the Taiwanese population [11]. Therefore, this retrospective and longitudinal cohort study was performed to examine the health status of fishery workers based on the most common causes of death and to determine whether they tended to have a rising incidence threat of developing some diseases compared with farmers and employed workers with similar socioeconomic status.

## 2. Materials and Methods

### 2.1. Source of Data

The Longitudinal Health Insurance Database 2000 (LHID2000), a subset of the Taiwan National Health Insurance Research Database (NHIRD), was used for this retrospective cohort study [12]. The LHID2000 collects claims data of >23 million people, representing >99.8% of Taiwanese residents. The LHID2000 contains claims data between 1997 and 2012 from 1 million random people who were alive on 1 July 2000. The representation of the general Taiwanese population for LHID2000 was validated by the Taiwan National Health Research Institutes [12]. The International Classification of Diseases, Ninth Revision, Clinical Modification (ICD-9-CM) codes were used for clinical diagnosis. More details on the NHIRD have been described previously [13,14,15]. In spite of the fact that secondary and de-identified data were used, the study has been approved by the Institutional Review Board (IRB No. 202002186B0).

### 2.2. Subjects and Covariates

Adult residents were included in this study. Information on occupation was available in the Registry for Beneficiaries sub-database of LHID2000. Only fishery workers (employed or small-scale workers in aquaculture, coastal, offshore, and marine settings), farmers (agriculture and small-scale workers), and employed workers (not owners) were included. Although the working environment and situation in coastal, offshore, and marine settings vary among fishery workers, most fishery workers are involved in aquafarming near inland or coastal Taiwan [1]. The demographic variables comprised gender, age, region of residence, urbanization level, and monthly income on 1 July 2000. The township of residence was classified into seven urbanization levels according to a previous survey [16]. The living region was categorized as north, central, south, and east/off islands. The monthly income was classified as <600, 600–800, and >800 USD. Information on living regions and monthly income were also available in the Registry for Beneficiaries sub-database.

### 2.3. Outcomes and Follow-Up

Based on the majority of causes of death in the Taiwanese population and previous literature related to the health of fishery workers [11,17,18,19], 18 health outcomes (newly diagnosed diseases) were selected and classified into cardiometabolic diseases, mental illness, infectious diseases, chronic kidney diseases (CKD), chronic obstructive pulmonary disease (COPD), fracture, and malignancy. Cardiometabolic diseases contained hypertension (ICD-9-CM: 401–405), coronary arterial disease (ICD-9-CM: 410–414), hospitalization for myocardial infarction (ICD-9-CM: 410), stroke (ICD-9-CM: 430–437), heart failure (ICD-9-CM: 428), diabetes (ICD-9-CM: 250), and dyslipidemia (ICD-9-CM: 272). Mental health-related illnesses consisted of anxiety (ICD-9-CM: 300), depression (ICD-9-CM: 296.2, 296.3, 648.44, 780.79), and peptic ulcer (ICD-9-CM: 531). Infectious diseases included hepatitis B and C virus infection (ICD-9-CM: 070.20, 070.22, 070.54, and V02.62) and tuberculosis (ICD-9-CM: 010–018). The ICD-9-CM codes used for the diagnosis of CKD; COPD; fracture; and malignancy (including hepatocellular carcinoma) were 580–589; 491, 492, and 496; 805–829; and 140–208 (155), respectively.

Adult residents with an old diagnosis of these diseases were considered event-free cases and were excluded from the analysis. The event of myocardial infarction, stroke, or heart failure was interpreted as an episode of hospitalization. The incident of fracture was interpreted as a visit to the emergency department or hospitalization. The diagnosis of malignancy (including hepatocellular carcinoma) was confirmed by the evidence of a critical illness certificate. The incident of other comorbidities (e.g., hypertension and diabetes) was classified as having at least two outpatient or inpatient diagnoses. Each participant was followed up from 1 July 2000 to the outcome occurrence and the withdrawal date from the National Health Insurance program, or 31 December 2012, whichever occurred first.

### 2.4. Data Analysis

Due to some differences in the baseline demographics among the groups (fishery workers, farmers, and employed workers), a cohort study with propensity score-matched to differentiate the outcomes among the groups was conducted. The predicted probability of being in one group was the propensity score (e.g., fishery workers), given some values of independent variables using multivariable logistic regression analysis. The propensity score was measured by age, gender, urbanization level, region of residence, and monthly income. The matching was handled using an acquisitive closest-neighbor arithmetic with a caliper of 0.2 times the standard bias of the logit of the propensity score, with a stochastic accordance order and without substitute. The balance between groups was assessed using the absolute value of the standardized difference (STD), where a value < 0.1 was considered no difference.

The incidence of newly diagnosed comorbidities was expressed as the incidence density, which denotes the figure of incidents per 1000 person-years. The occurrence of newly diagnosed comorbidities between the groups in the propensity score-matched cohort study was differentiated using the Cox proportional model. The study group was the only descriptive element in the Cox proportional hazards model. The potential outcome dependency between two subjects in the same matching pair was accommodated using a robust standard error recognized as the marginal model. A two-sided *p* < 0.05 was defined as statistically significant. We used SAS version 9.4 (SAS Institute, Cary, NC, USA) in this study.

## 3. Results

### 3.1. Identification of the Study Participants

**Figure 1:**
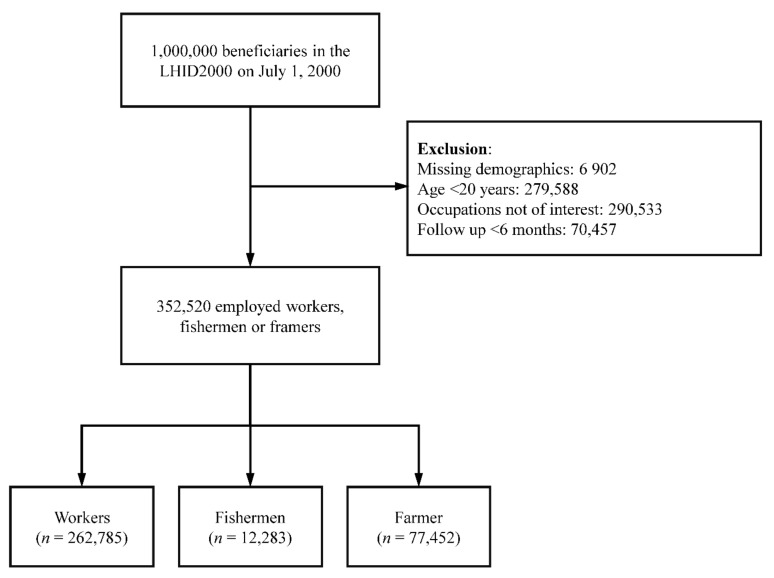
Inclusion criteria.

Figure 1 shows the study inclusion process. Among the 1 million beneficiaries in the LHID2000 on 1 July 2000, participants with missing demographics (*n* = 6902), those aged <20 years (*n* = 279,588), those engaged in occupations that were not of interest (other than employed workers, fishery workers, and farmers; *n* = 290,533), and those who were followed for <6 months (*n* = 70,457) were excluded. In total, 352,520 adults engaged in the occupations of interest were included—12,283 fishery workers, 77,452 farmers, and 262,785 employed workers.

### 3.2. Newly Diagnosed Comorbidities in Fishery Workers Compared to Those in Farmers

Table 1 lists the baseline demographics of fishery workers and farmers before and after propensity score matching. Before matching, fishery workers were much younger than farmers (42.2 vs. 58.5 years, STD = −1.09). The urbanization level of residents was lower among the farmers, especially in the lowest three levels (levels 5–7). Fishery workers were distributed mainly in southern Taiwan (52.9%), while farmers were distributed equally across Taiwan. Both fishery workers and farmers had low monthly incomes. The average follow-up was substantially briefer for farmers than for fishery workers (10.1 vs. 11.7 years). After matching, there were negligible distinctions in the basic demographics between fishery workers and farmers, with all absolute STD values < 0.1 (Table 1).

The risk of newly diagnosed comorbidities between fishery workers and farmers was compared in the matched cohort (Table 2). Compared to farmers, fishery workers had significantly greater risks for 11 comorbidities—hypertension (hazard ratio [HR]: 1.09, 95% confidence interval [CI]: 1.03–1.14), diabetes (HR: 1.15, 95% CI: 1.08–1.23), dyslipidemia (HR: 1.13, 95% CI: 1.07–1.19), depression (HR: 1.15, 95% CI: 1.07–1.24), peptic ulcer (HR: 1.10, 95% CI: 1.04–1.15), hepatitis B virus infection (HR: 1.26, 95% CI: 1.13–1.40), hepatitis C virus infection (HR: 1.31, 95% CI: 1.14–1.50), CKD (HR: 1.11, 95% CI: 1.02–1.20), COPD (HR: 1.10, 95% CI: 1.02–1.19), any malignancy (HR: 1.26, 95% CI: 1.13–1.40), and hepatocellular carcinoma (HR: 1.57, 95% CI: 1.22–2.04).

### 3.3. Newly Diagnosed Comorbidities in Fishery Workers Compared to Those in Employed Workers

Table 3 lists the baseline demographics of the fishery and employed workers before and after propensity score matching. Before matching, fishery workers were older than employed workers (42.2 vs. 35.5 years, STD = 0.57). The urbanization level of the residents was lower among fishery workers, especially in the highest two levels (levels 1–2). Fishery workers were distributed mainly in southern Taiwan (52.9%), while employed workers were distributed mainly in northern Taiwan (69.6%). All fishery workers had a monthly income of <800 USD, whereas nearly half of employed workers (44.7%) had a monthly income of >800 USD. After matching, there were negligible distinctions in the basic demographics between fishery workers and employed workers, with all absolute STD values < 0.1 (Table 3).

The risk of newly diagnosed comorbidities between fishery workers and employed workers was compared in the matched cohort (Table 4). Compared to employed workers, fishery workers had significantly greater risks for 14 comorbidities—hypertension (HR: 1.11, 95% CI: 1.04–1.17), coronary arterial disease (HR: 1.18, 95% CI: 1.09–1.28), diabetes (HR: 1.21, 95% CI: 1.12–1.31), dyslipidemia (HR: 1.18, 95% CI: 1.11–1.25), anxiety (HR: 1.08, 95% CI: 1.01–1.16), depression (HR: 1.38, 95% CI: 1.27–1.51), peptic ulcer (HR: 1.17, 95% CI: 1.11–1.24), hepatitis B virus infection (HR: 1.32, 95% CI: 1.17–1.49), hepatitis C virus infection (HR: 2.06, 95% CI: 1.71–2.47), CKD (HR: 1.16, 95% CI: 1.06–1.27), COPD (HR: 1.17, 95% CI: 1.07–1.28), fracture (HR: 1.11, 95% CI: 1.01–1.21), any malignancy (HR: 1.21, 95% CI: 1.07–1.37), and hepatocellular carcinoma (HR: 1.67, 95% CI: 1.24–2.26).

## 4. Discussion

This is the largest retrospective and longitudinal study to show the incidence rate of newly diagnosed comorbidities among fishery workers, farmers, and employed workers. After using the propensity score-matched cohort and applying the Cox proportional hazards model, this study showed three important findings. First, fishery workers had significantly greater risks for 11 newly diagnosed diseases than farmers. Second, fishery workers had significantly greater risks for 14 newly diagnosed diseases than employed workers. Third, mental health-related illnesses, such as anxiety, depression, and peptic ulcers, must be noted among fishery workers.

The present study showed that hepatocellular carcinoma had a dominant incidence rate in fishery workers compared to that in farmers and employed workers. However, in some previous studies in Brazil and Nordic countries [20,21], skin and lip cancer were more common in seafarers and fishermen than in the general population. A possible reason might be the difference in the working environment and situation, as previous studies included both maritime populations, but our study mainly included small-scale aquaculture farming workers [1]. Moreover, in our study, the ID and HR of hepatitis B and C virus infections were more critical in fishery workers than in farmers and employed workers. Based on current evidence, hepatitis B and C viruses are the major causes of liver fibrosis and progression to hepatocellular carcinoma worldwide [22,23]. This explains why fishery workers had significantly greater risks for malignancy and hepatocellular carcinoma than farmers and employed workers in our study.

Globally, more than 325 million people are infected with hepatitis B and C virus [23]. Mass infant vaccination has significantly controlled hepatitis B virus infection; however, no vaccine has been developed to prevent hepatitis C virus infection. Hepatitis C virus is a blood transmission disease, which is most prevalently conveyed through exposure to injection drugs, insufficient disinfection of medical devices, or blood products [23,24]. Further, >50% of people infected with chronic hepatitis C virus (CHC) generally develop long-term effects. Owing to the inflammation and infection mechanism, some previous studies have shown that patients with CHC infection have a trend relation with type 2 diabetes and magnified cardiovascular diseases compared with those without hepatitis C virus infection [25,26,27]. In addition, many patients with CHC infection also develop extrahepatic health problems, such as diabetes mellitus, heart disease, hypertension, stroke, and renal impairment [26,27].

The treatment of hepatitis C virus infection has advanced from interferon-based therapy to direct-acting antivirals (DAAs) [24]. Antiviral treatment for hepatitis C virus infection has been shown to be successful. Lately, DAAs such as asunaprevir/daclatasvir, elbasvir/grazoprevir, and ledipasvir/sofosbuvir have enhanced cure rates with >95% efficiency, few side effects, and a short treatment time of 2–3 months [24,28,29]. Furthermore, DAA treatment is beneficial for hepatitis C virus clearance and is associated with better cardiometabolic function, including mitigated fasting glucose and glycated hemoglobin levels [26,27]. Eradication of the hepatitis C virus infection in 2030 has been announced by the World Health Organization [23], and the Taiwan Department of Health has set a goal for hepatitis C elimination by 2025 with free DAA treatment [24].

However, information on the benefits of free DAA treatment might be a barrier for fishery workers because of their resource-limited working and living environment. Our previous studies found that many adults with CHC live in countryside areas and have less literacy of hepatitis, unhealthy lifestyles, and abnormal kidney and liver function [19,28]. Hence, primary healthcare providers must provide accessibility and initiate an interdisciplinary collaborative approach to reduce the rising burden of liver cancer and its extrahepatic manifestations, such as cardiovascular diseases, diabetes, and renal diseases. Moreover, advocating for fishery workers to receive antiviral treatment via health agencies in the public and private sectors is necessary. For instance, it is necessary to educate people on new information related to free antiviral treatment, reduce the obstacle of work–time collision, and use the proper mass media to enhance health information and hospital referrals through interdisciplinary collaboration [19,28].

Our study indicated that fishery workers had a higher incidence density rate of cardiometabolic diseases and psychological distress, including hypertension, coronary arterial disease, diabetes, dyslipidemia, anxiety, and depression, than farmers and employed workers. These results are consistent with those of some previous observational findings showing cardiovascular diseases as the major cause of mortality in fishery workers onboard a ship in coastal environments with varying temperatures [9,30]. Cardiometabolic diseases are associated with some specific risk factors or metabolic syndrome before disease diagnosis, e.g., elevated blood pressure, serum glucose levels, fasting blood lipid levels, and central obesity [31,32]. These abnormal biomarkers are significantly associated with inactivity, unhealthy diets, and substance use [31,32,33]. Therefore, early detection of metabolic syndrome for these populations was an important issue for all local health agencies.

Although our study did not explore health-related behaviors owing to working settings, some previous cross-sectional studies pointed out that an unhealthy lifestyle in fishery workers, including an advanced prevalence of smoking and alcohol consumption and a lack of adequate vegetables and fruits in the dietary intake [34,35]. Most cardiometabolic diseases can be avoided by reducing risky behaviors such as cigarette smoking, unhealthy eating, sedentary lifestyle, and alcohol consumption [31,32,33]. Hence, further studies should detect cardiometabolic risk factors and diseases as early as possible in fishery workers to initiate management with counseling and medications, and prevent psychological distress via workplace health promotion. In addition, it is necessary to perform a deep exploration via qualitative research design to understand fishery workers’ lifestyle pattern, e.g., how they live 24 h a day, and what they eat throughout a week.

There are some limitations to the study. First, the nationwide database did not provide data on disease seriousness; laboratory information; body mass index; or personal behaviors, such as tobacco use, alcohol consumption, adopting a balanced diet, and physical activity. Second, although Taiwan National Health Insurance was initialed in 1995, the healthcare delivery system or medical resources might have been limited in rural areas. Therefore, the incidence rate of disease diagnosis might be underestimated in the study. Third, there is a time gap between the current study (2000–2012) and the present year of 2022. Some demographic data, such as the status of monthly income and urbanization level, are different between decades. Future studies utilizing more recent data are encouraged.

There are some strengths in the study in terms of a comprehensive understanding of the health status of fishery workers. First, a nationwide database was used to allow us to explore a great sample of patients for more than a 13-year consecutive period. Second, because the NHIRD offered disease information from Taiwanese people overall, the effects of deviation related to data collection, region, and institution were minimized. Third, the NHIRD eliminated the need to minimize the number of patients in the cohort who strayed from consecutive progress and empowered us to capture large, geographically scattered samples of patients with diversified sociodemographic features. Fourth, important strengths include the national population-based and retrospective longitudinal cohort study design.

## 5. Conclusions

In conclusion, our findings demonstrate that fishery workers were more impacted by cardiometabolic diseases, infectious diseases, mental distress, and malignancy, especially liver cancer, than farmers and employed workers. The present findings can be used to tackle health inequities that are unfair, avoidable, or remediable [36]. Although the Taiwan government launched the National Health Insurance, which covered 99% of the residents and received a great scale of contentment, health facility density and access to clinics or hospitals for treatments are limited because most fishery workers live around the coastal area. Further studies must investigate social inequality and conduct health equity strategies through public health actions. Additionally, these findings highlight the need to initiate culture-tailored health promotion programs and advance primary healthcare services at the nationwide level.

## Figures and Tables

**Table 1:** Baseline demographics of the fishery workers and farmers before and after propensity score matching.

Variable	Before Matching			After Matching		
	Fishery (*n* = 12,283)	Farmer (*n* = 77,452)	STD	Fishery (*n* = 12,160)	Farmer (*n* = 12,160)	STD
Age, year	42.2 ± 12.8	58.5 ± 16.8	−1.09	42.2 ± 12.8	42.1 ± 12.5	0.01
Male sex	6442 (52.4)	39,666 (51.2)	0.02	6365 (52.3)	6366 (52.4)	<0.01
Age group, year						
20–29	2434 (19.8)	4333 (5.6)	0.44	2391 (19.7)	2319 (19.1)	0.01
30–39	3448 (28.1)	9759 (12.6)	0.39	3408 (28.0)	3479 (28.6)	−0.01
40–49	3117 (25.4)	10,909 (14.1)	0.29	3093 (25.4)	3188 (26.2)	−0.02
50–59	1937 (15.8)	11,254 (14.5)	0.03	1924 (15.8)	1954 (16.1)	−0.01
60–69	1137 (9.3)	19,792 (25.6)	−0.44	1135 (9.3)	1050 (8.6)	0.02
≥70	210 (1.7)	21,405 (27.6)	−0.79	209 (1.7)	170 (1.4)	0.03
Urbanization level of the residence						
Level 1: the highest	1033 (8.4)	5322 (6.9)	0.06	1026 (8.4)	1031 (8.5)	<0.01
Level 2	1903 (15.5)	14,249 (18.4)	−0.08	1892 (15.6)	1922 (15.8)	−0.01
Level 3	2095 (17.1)	11,994 (15.5)	0.04	2081 (17.1)	2013 (16.6)	0.01
Level 4	6494 (52.9)	24,803 (32.0)	0.43	6424 (52.8)	6461 (53.1)	−0.01
Level 5	0 (0.0)	3613 (4.7)	−0.31	0 (0.0)	0 (0.0)	<0.01
Level 6	169 (1.4)	8259 (10.7)	−0.40	169 (1.4)	168 (1.4)	<0.01
Level 7: the lowest	589 (4.8)	9212 (11.9)	−0.26	568 (4.7)	565 (4.6)	<0.01
Region of the residence						
North	2936 (23.9)	19,571 (25.3)	−0.03	2918 (24.0)	2953 (24.3)	−0.01
Central	2095 (17.1)	11,994 (15.5)	0.04	2081 (17.1)	2013 (16.6)	0.01
South	6494 (52.9)	28,416 (36.7)	0.33	6424 (52.8)	6461 (53.1)	−0.01
East/off island	758 (6.2)	17,471 (22.6)	−0.48	737 (6.1)	733 (6.0)	<0.01
Monthly income, USD						
<600	565 (4.6)	2085 (2.7)	0.10	471 (3.9)	311 (2.6)	0.07
600–800	11,718 (95.4)	75,367 (97.3)	−0.10	11,689 (96.1)	11,849 (97.4)	−0.07
>800	0 (0.0)	0 (0.0)	<0.01	0 (0.0)	0 (0.0)	<0.01
Follow-up year	11.7 ± 2.5	10.1 ± 4.2	0.47	11.7 ± 2.5	11.7 ± 2.7	0.02

STD, standardized difference; USD, United States Dollar; Data were presented as frequency (percentage) or mean ± standard deviation.

**Table 2:** The incidence of newly diagnosed comorbidities between the fishery workers and farmers.

Comorbidity Condition	Fishery (*n* = 12,160)		Farmer (*n* = 12,160)		HR (95% CI) of Fisherman	*p* Value
	Event No. (%)	ID (95% CI) #	Event No. (%)	ID (95% CI) #		
Cardiometabolic diseases						
Hypertension	2932 (24.1)	23.5 (22.7–24.4)	2708 (22.3)	21.6 (20.8–22.5)	1.09 (1.03–1.14)	0.001
Coronary arterial disease	1541 (12.7)	11.5 (11.0–12.1)	1484 (12.2)	11.2 (10.6–11.8)	1.03 (0.96–1.10)	0.386
Myocardial infarction	130 (1.1)	0.91 (0.76–1.07)	118 (1.0)	0.83 (0.68–0.98)	1.10 (0.86–1.41)	0.462
Stroke	137 (1.1)	0.96 (0.80–1.12)	119 (1.0)	0.84 (0.69–0.99)	1.15 (0.90–1.47)	0.277
Heart failure	234 (1.9)	1.7 (1.4–1.9)	201 (1.7)	1.4 (1.2–1.6)	1.16 (0.96–1.40)	0.118
Diabetes	1721 (14.2)	13.0 (12.3–13.6)	1496 (12.3)	11.2 (10.7–11.8)	1.15 (1.08–1.23)	<0.001
Dyslipidemia	2800 (23.0)	22.3 (21.5–23.2)	2497 (20.5)	19.7 (19.0–20.5)	1.13 (1.07–1.19)	<0.001
Mental illness						
Anxiety	2070 (17.0)	16.2 (15.5–16.9)	2066 (17.0)	16.2 (15.5–16.9)	1.00 (0.94–1.06)	0.949
Depression	1462 (12.0)	10.9 (10.3–11.4)	1273 (10.5)	9.4 (8.9–10.0)	1.15 (1.07–1.24)	<0.001
Peptic ulcer	3112 (25.6)	25.6 (24.7–26.5)	2857 (23.5)	23.4 (22.5–24.2)	1.10 (1.04–1.15)	0.001
Infectious disease						
Hepatitis B virus	748 (6.2)	5.4 (5.0–5.8)	595 (4.9)	4.3 (3.9–4.6)	1.26 (1.13–1.40)	<0.001
Hepatitis C virus	468 (3.8)	3.3 (3.0–3.6)	358 (2.9)	2.6 (2.3–2.8)	1.31 (1.14–1.50)	<0.001
Tuberculosis	183 (1.5)	1.3 (1.1–1.5)	180 (1.5)	1.3 (1.1–1.5)	1.01 (0.82–1.24)	0.913
Chronic kidney disease	1222 (10.0)	9.0 (8.5–9.5)	1104 (9.1)	8.1 (7.7–8.6)	1.11 (1.02–1.20)	0.014
COPD	1298 (10.7)	9.7 (9.1–10.2)	1177 (9.7)	8.8 (8.3–9.3)	1.10 (1.02–1.19)	0.015
Fracture	1258 (10.3)	9.3 (8.8–9.8)	1234 (10.1)	9.1 (8.6–9.6)	1.02 (0.94–1.10)	0.669
Malignancy	747 (6.1)	5.3 (4.9–5.7)	590 (4.9)	4.2 (3.9–4.5)	1.26 (1.13–1.40)	<0.001
Hepatocellular carcinoma	148 (1.2)	1.04 (0.87–1.21)	93 (0.8)	0.66 (0.52–0.79)	1.57 (1.22–2.04)	<0.001

ID, incidence density; HR, hazard ratio; CI, confidence interval; # Number of incident events per 1000 person-years; COPD, chronic obstructive pulmonary disease.

**Table 3:** Baseline demographics of the fishery and employed workers before and after propensity score matching.

Variable	Before Matching			After Matching		
	Fishery (*n* = 12,283)	Worker (*n* = 262,785)	STD	Fishery (*n* = 10,489)	Worker (*n* = 10,489)	STD
Age, year	42.2 ± 12.8	35.5 ± 10.3	0.57	40.1 ± 12.0	40.3 ± 12.3	−0.01
Male sex	6442 (52.4)	146,837 (55.9)	−0.07	5429 (51.8)	5614 (53.5)	−0.04
Age group, year						
20–29	2434 (19.8)	94,057 (35.8)	−0.36	2434 (23.2)	2453 (23.4)	<0.01
30–39	3448 (28.1)	88,398 (33.6)	−0.12	3223 (30.7)	3190 (30.4)	0.01
40–49	3117 (25.4)	55,721 (21.2)	0.10	2676 (25.5)	2662 (25.4)	<0.01
50–59	1937 (15.8)	18,823 (7.2)	0.27	1399 (13.3)	1355 (12.9)	0.01
60–69	1137 (9.3)	4705 (1.8)	0.33	635 (6.1)	678 (6.5)	−0.02
≥70	210 (1.7)	1081 (0.4)	0.13	122 (1.2)	151 (1.4)	−0.02
Urbanization level of the residence						
Level 1: the highest	1033 (8.4)	79,628 (30.3)	−0.58	1029 (9.8)	1080 (10.3)	−0.02
Level 2	1903 (15.5)	103,200 (39.3)	−0.55	1896 (18.1)	2073 (19.8)	−0.04
Level 3	2095 (17.1)	28,377 (10.8)	0.18	1969 (18.8)	2123 (20.2)	−0.04
Level 4	6494 (52.9)	31,517 (12.0)	0.97	4883 (46.6)	4528 (43.2)	0.07
Level 5	0 (0.0)	429 (0.2)	−0.06	0 (0.0)	0 (0.0)	<0.01
Level 6	169 (1.4)	934 (0.4)	0.11	128 (1.2)	105 (1.0)	0.02
Level 7: the lowest	589 (4.8)	18,700 (7.1)	−0.10	584 (5.6)	580 (5.5)	<0.01
Region of the residence						
North	2936 (23.9)	182,828 (69.6)	−1.03	2925 (27.9)	3153 (30.1)	−0.05
Central	2095 (17.1)	28,377 (10.8)	0.18	1969 (18.8)	2123 (20.2)	−0.04
South	6494 (52.9)	31,946 (12.2)	0.97	4883 (46.6)	4528 (43.2)	0.07
East/off island	758 (6.2)	19,634 (7.5)	−0.05	712 (6.8)	685 (6.5)	0.01
Monthly income, USD						
<600	565 (4.6)	105,120 (40.0)	−0.94	565 (5.4)	468 (4.5)	0.04
600–800	11,718 (95.4)	40,102 (15.3)	2.72	9924 (94.6)	10,021 (95.5)	−0.04
>800	0 (0.0)	117,563 (44.7)	−1.27	0 (0.0)	0 (0.0)	<0.01
Follow-up year	11.7 ± 2.5	10.8 ± 4.1	0.28	11.8 ± 2.4	11.2 ± 3.5	0.22

STD, standardized difference; USD, United States Dollar; Data were presented as frequency (percentage) or mean ± standard deviation.

**Table 4:** The incidence of newly diagnosed comorbidities between the fishery workers and employed workers.

Comorbid Condition	Fishery (*n* = 10,489)		Employed Worker (*n* = 10,489)		HR (95% CI) of Fisherman	*p* Value
	Event No. (%)	ID (95% CI) #	Event No. (%)	ID (95% CI) #		
Cardiometabolic diseases						
Hypertension	2360 (22.5)	21.5 (20.6–22.4)	2035 (19.4)	19.4 (18.6–20.3)	1.11 (1.04–1.17)	0.001
Coronary arterial disease	1172 (11.2)	10.0 (9.4–10.6)	943 (9.0)	8.5 (7.9–9.0)	1.18 (1.09–1.28)	<0.001
Myocardial infarction	96 (0.92)	0.78 (0.62–0.93)	99 (0.94)	0.85 (0.68–1.01)	0.91 (0.69–1.21)	0.527
Stroke	106 (1.01)	0.86 (0.69–1.02)	100 (0.95)	0.86 (0.69–1.02)	1.00 (0.76–1.31)	1.000
Heart failure	161 (1.5)	1.3 (1.1–1.5)	141 (1.3)	1.2 (1.0–1.4)	1.08 (0.86–1.34)	0.516
Diabetes	1375 (13.1)	11.8 (11.2–12.5)	1088 (10.4)	9.8 (9.2–10.4)	1.21 (1.12–1.31)	<0.001
Dyslipidemia	2269 (21.6)	20.6 (19.7–21.4)	1855 (17.7)	17.5 (16.7–18.3)	1.18 (1.11–1.25)	<0.001
Mental illness						
Anxiety	1703 (16.2)	15.2 (14.5–15.9)	1498 (14.3)	14.0 (13.3–14.7)	1.08 (1.01–1.16)	0.025
Depression	1242 (11.8)	10.6 (10.0–11.2)	864 (8.2)	7.7 (7.2–8.2)	1.38 (1.27–1.51)	<0.001
Peptic ulcer	2605 (24.8)	24.5 (23.6–25.5)	2141 (20.4)	20.9 (20.0–21.8)	1.17 (1.11–1.24)	<0.001
Infectious diseases						
Hepatitis B virus	648 (6.2)	5.4 (5.0–5.8)	466 (4.4)	4.1 (3.7–4.5)	1.32 (1.17–1.49)	<0.001
Hepatitis C virus	357 (3.4)	2.9 (2.6–3.2)	165 (1.6)	1.4 (1.2–1.6)	2.06 (1.71–2.47)	<0.001
Tuberculosis	141 (1.34)	1.1 (1.0–1.3)	139 (1.33)	1.2 (1.0–1.4)	0.96 (0.76–1.21)	0.708
Chronic kidney disease	957 (9.1)	8.1 (7.6–8.6)	786 (7.5)	7.0 (6.5–7.5)	1.16 (1.06–1.27)	0.002
COPD	997 (9.5)	8.5 (8.0–9.0)	808 (7.7)	7.2 (6.7–7.7)	1.17 (1.07–1.28)	0.001
Fracture	1036 (9.9)	8.8 (8.2–9.3)	887 (8.5)	7.9 (7.4–8.4)	1.11 (1.01–1.21)	0.025
Malignancy	564 (5.4)	4.6 (4.2–5.0)	446 (4.3)	3.9 (3.5–4.2)	1.21 (1.07–1.37)	0.002
Hepatocellular carcinoma	112 (1.07)	0.90 (0.74–1.07)	62 (0.59)	0.53 (0.40–0.66)	1.67 (1.24–2.26)	<0.001

ID, incidence density; HR, hazard ratio; CI, confidence interval; # Number of incident events per 1000 person-years; COPD, chronic obstructive pulmonary disease.

## Data Availability

The data used to support the findings of this study are available from the corresponding author upon request.

## Abbreviations

NHIRD:National Health Insurance Research Database; LHID, Longitudinal Health Insurance Database; ICD-9-CM, International Classification of Diseases, Ninth Revision, Clinical Modification; CKD, chronic kidney diseases; STD, standardized difference; USD, United States Dollar; ID, incidence density; HR, hazard ratio; CI, confidence interval; COPD, chronic obstructive pulmonary disease; CHC, chronic hepatitis C virus; DAAs, direct-acting antivirals.
